# Uncommon opsin’s retinal isomer is involved in mammalian sperm thermotaxis

**DOI:** 10.1038/s41598-024-61488-3

**Published:** 2024-05-10

**Authors:** Alexander Brandis, Debarun Roy, Ishita Das, Mordechai Sheves, Michael Eisenbach

**Affiliations:** 1 Department of Life Sciences Core Facilities, The Weizmann Institute of Science, 7610001 Rehovot, Israel; 2 Department of Biomolecular Sciences, The Weizmann Institute of Science, 7610001 Rehovot, Israel; 3 Department of Molecular Chemistry and Materials Science, The Weizmann Institute of Science, 7610001 Rehovot, Israel; 4 Present Address: Department of Neuroscience, Developmental and Regenerative Biology, The University of Texas at San Antonio, San Antonio, TX 78249 USA

**Keywords:** Sperm retinal, Thermosensor, Sperm thermotaxis, Tri-*cis* retinal, Thermosensing, Vitamin A starvation, G protein-coupled receptors, Biochemistry, Proteins

## Abstract

In recent years it became apparent that, in mammals, rhodopsin and other opsins, known to act as photosensors in the visual system, are also present in spermatozoa, where they function as highly sensitive thermosensors for thermotaxis. The intriguing question how a well-conserved protein functions as a photosensor in one type of cells and as a thermosensor in another type of cells is unresolved. Since the moiety that confers photosensitivity on opsins is the chromophore retinal, we examined whether retinal is substituted in spermatozoa with a thermosensitive molecule. We found by both functional assays and mass spectrometry that retinal is present in spermatozoa and required for thermotaxis. Thus, starvation of mice for vitamin A (a precursor of retinal) resulted in loss of sperm thermotaxis, without affecting motility and the physiological state of the spermatozoa. Thermotaxis was restored after replenishment of vitamin A. Using reversed-phase ultra-performance liquid chromatography mass spectrometry, we detected the presence of retinal in extracts of mouse and human spermatozoa. By employing UltraPerformance convergence chromatography, we identified a unique retinal isomer in the sperm extracts—tri-*cis* retinal, different from the photosensitive 11-*cis* isomer in the visual system. The facts (a) that opsins are thermosensors for sperm thermotaxis, (b) that retinal is essential for thermotaxis, and (c) that tri*-cis* retinal isomer uniquely resides in spermatozoa and is relatively thermally unstable, suggest that tri*-cis* retinal is involved in the thermosensing activity of spermatozoa.

## Introduction

Opsins are common in a wide variety of species, from bacteria and archaea to mammals. In mammals, they are G-protein-coupled receptors, well known for their function as photosensors in vision^1–3^. Several years ago, this classical role of opsins had a twist when rhodopsin (Opsin-2) was discovered to act as a thermosensor in Drosophila larvae^4^, and when opsins were found to be present in mammalian spermatozoa and act there as thermosensors for thermotaxis^5^. This twist has raised the intriguing question of how a given opsin, rhodopsin for instance, which is structured to be a highly efficient photosensor in the visual system, is modified in spermatozoa to become a thermosensor.

The moiety that confers photosensitivity on opsins is the chromophore retinal, bound to the protein via a protonated Schiff base at a conserved lysine residue. In the case of human opsins, the retinal undergoes light-stimulated isomerization from 11-*cis*-retinal to all *trans*-retinal, and the protein undergoes a conformational change and interacts with the G-protein transducin (for a review, see^6^). It is reasonable to assume that when an opsin acts as a thermosensor rather than a photosensor, its photosensitive chromophore would be substituted for a thermosensitive group. However, hydroxylamine, which competes with the lysine residue and reacts with the retinal chromophore, was reported to inhibit human sperm thermotaxis, suggesting that retinal may be involved in thermosensing as well^5^. Yet, retinal could not be detected by mass spectrometry (MS) in human spermatozoa^5^. The aim of the current study was to determine whether retinal is present in mammalian spermatozoa and involved in thermosensing, and if so—in what way it is different from visual retinal.

## Results

### Vitamin A is essential for sperm thermotaxis

The chromophore retinal is the aldehyde form of vitamin A. To determine whether vitamin A or a derivative thereof is involved in sperm thermotaxis, we examined whether starving mice for vitamin A would affect this process. For measuring thermotaxis, we employed earlier-described^7,8^ two-compartment thermoseparation tubes, found adequate to the limited number of spermatozoa that can be isolated from the mouse epididymis^5^. Each thermotaxis assay consisted of pairs of thermoseparation tubes (internal diameter 3.8 mm), one tube subjected to a temperature gradient (from 35 to 37 °C, termed ‘the gradient tube’) and one to a constant temperature (35 °C, termed ‘no-gradient control’)^7,8^. Spermatozoa were added to one of the compartments of the thermoseparation tube and the number of spermatozoa accumulating in the other compartment was compared between the gradient tube and the no-gradient control. In such assays, an excess of sperm accumulation in the warmer compartment of the gradient tube relative to the parallel compartment of the no-gradient control faithfully reflects thermotaxis. This has been well established in earlier studies by a variety of means^7,8^. Because spermatozoa must be capacitated for being thermotactically responsive^9^, we performed all assays with sperm samples that had been incubated under capacitating conditions.

**Figure 1:**
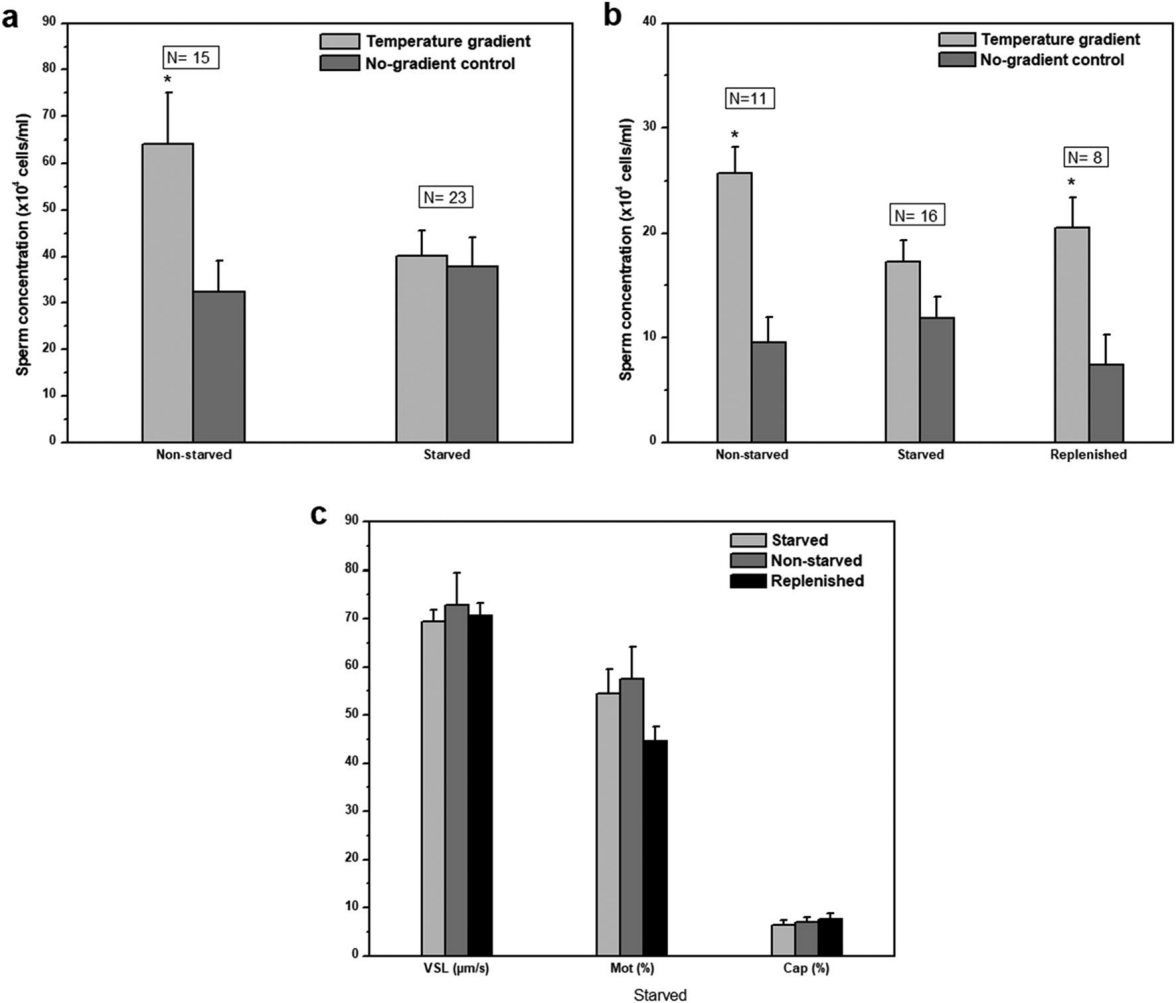
Effect of vitamin A-starvation on mouse sperm thermotaxis. (**a**) Sperm thermotaxis of starved mice. All mice were 4-weeks old at the beginning of the starvation. The columns stand for the accumulation of spermatozoa in the warmer compartment, using the two-compartment separation tube^7,8^ (mean ± SEM). All values were normalized according to the average number of spermatozoa in the cold chamber (12.8 × 10^6^ cells). N is the number of mice tested. *P = 0.0005 according to Linear mixed effects model, with treatment and gradient as fixed factors, and mouse ID as a random factor. No significant difference was observed in the group of starved mice (*P* = 0.98). (**b**) Sperm thermotaxis of starved and replenished mice. All mice were 12-weeks old at the beginning of the experiment. The columns stand for the accumulation of spermatozoa in the warmer compartment (mean ± SEM). All values were normalized according to the average number of spermatozoa in the cold chamber (9.4 × 10^6^ cells). N is the number of mice tested. *P < 0.002 for the difference between gradient and the no-gradient control according to Linear mixed effects model, with treatment and gradient as fixed factors, and mouse ID as a random factor. Post-hoc comparisons were done using Tukey’s test. The statistical analyses were carried out using the R packages ‘lme4’, ‘lmerTest’ and ‘lsmeans’. The difference between the sperm concentrations in panel a and b is probably due to the younger age of the mice in panel a than those in panel b, resulting in their higher motility. This higher motility in a is reflected in the higher sperm concentration in the columns of no-gradient control in panel a relative to b. The difference did not affect the result of the thermotaxis assay because each assay had its own no-gradient control from the very same sample. (**c**) Capacitation and motility of the sperm samples measured in panel b. Note that the Y axis has a different meaning for each group of columns, as written below the groups. The values shown are the mean ± SEM of 8, 7 and 8 starved, non-starved and replenished mice, respectively (capacitation) or of 10, 8 and 9 starved, non-starved and replenished mice (motility). [The motility parameters and capacitation level of the non-starved group are also presented as wild-type mice in Roy et al*.*^10^ (Fig. 6b and Table S3 there)]. An insignificant difference between the groups (*P* > 0.05) was established by one-way ANOVA with Tukey–Kramer post-test using InStat statistical software.

First, we compared the thermotactic activity of spermatozoa from normally-fed mice (termed hereafter ‘non-starved mice’) with that of mice fed for 4–9 weeks with the same diet but with vitamin A omitted (termed ‘starved mice’). Both groups were 4 weeks old at the beginning of the experiment. The starvation apparently caused loss of difference between sperm accumulation in the gradient tube *versus* the no-gradient control, suggesting loss of thermotactic activity in the group of starved mice (Fig. 1a). To verify these results, we examined whether thermotactic activity can be restored upon vitamin A replenishment. We fed again one group of mice with a vitamin A-containing diet and another group with a vitamin A-deficient diet (both groups were 12 weeks old at the beginning of the experiment). After 4 weeks, when the starved mice lost thermotactic activity, we divided them into two subgroups. One subgroup continued to be starved for vitamin A for an additional period (up to 10 weeks total starvation time), whereas the other subgroup was switched to vitamin A-containing diet for the same period, along with additional oral administration of this vitamin (termed hereafter ‘replenished mice’). In other words, we compared between three groups of mice: A control group of non-starved mice on normal diet, a group of starved mice, and a group of starved mice whose spermatozoa stopped being thermotactic after 4 weeks of vitamin A starvation and then replenished with the vitamin (supplementary Figure S1 for a scheme). The accumulation of spermatozoa from the starved mice in the gradient tube was not significantly different from the no-gradient control (Fig. 1b; for being strict, we even included in this group mice starved for less than 4 weeks, and which were still thermotactically active). This indicated that spermatozoa of starved mice were thermotactically inactive. In contrast, the accumulation of spermatozoa from non-starved mice in the gradient tube was significantly higher than the no-gradient control (Fig. 1b). Likewise did spermatozoa from replenished mice (Fig. 1b). These results indicate that both groups, non-starved and replenished, were thermotactically active. The no-gradient control, which is only dependent on sperm motility, was similar in all three groups (Fig. 1b; *P* ≥ 0.8), indicating that the loss of thermotactic activity during the starvation for vitamin A was not due to some starvation effect on the motility of the spermatozoa. Furthermore, the measured motility parameters that might directly affect the accumulation in the warm compartment—the straight-linear velocity (VSL) and the percent of motile cells were similar in all three groups (Fig. 1c; Supplementary Table S1 for all measured motility parameters).

As another control, we also measured the capacitation level of the spermatozoa. This is because only capacitated spermatozoa are thermotactically responsive^9^ and because only a small fraction of the spermatozoa become capacitated at any given time when incubated under capacitating conditions^11^. This means that any effect of starvation on the capacitation level might affect the thermotactic response. Moreover, because capacitation reflects the sperm’s ability to fertilize an oocyte^12^ and because capacitation involves many biochemical and signaling processes^13^, measuring the effect of starvation on the capacitation level is essentially a measurement of the effect of starvation on the overall physiological state of the spermatozoa. Clearly, the capacitation level was similar under all conditions (Fig. 1c), suggesting that the effects of vitamin A-starvation on sperm accumulation in the warmer compartment (Fig. 1b) were true effects on thermotaxis.

Depletion of vitamin A from various types of single cells and cell cultures has been demonstrated to cause oxidative stress, mitochondrial dysfunction, and PARP-1-dependent energy deprivation^14^. Mitochondrial dysfunction could potentially impact sperm motility^15^ as well as the level of sperm capacitation^16^. Nonetheless, these effects are not anticipated to influence the conclusions derived from the thermotaxis assays. This is because a decrease in motility would similarly decrease the number of spermatozoa accumulating in the relevant compartment of both the gradient tube and the no-gradient control tube. Consequently, the determination of thermotaxis occurrence would not be affected. Moreover, the starvation did not impact sperm motility, as evident from the comparable values observed in the no-gradient control tubes under starvation and non-starvation conditions (Fig. 1a, b), along with the similarity in measured motility parameters between these conditions (Fig. 1c and Supplementary Table S1). As for the capacitation level of the spermatozoa, the direct measurement of this level demonstrated that it was similar in spermatozoa from starved and non-starved mice (Fig. 1c). Hence, neither the motility nor the levels of capacitated cells were affected by vitamin A starvation. This indicates that, under our experimental conditions, the mitochondria were normally functional. The preservation of mitochondrial function probably stems from the very slow, gradual, and probably incomplete vitamin A depletion in whole animals (like in our study), as opposed to the sudden and complete depletion seen in single cells and cell cultures^14^.

All the results above, taken together, suggest that retinal is involved in sperm thermotaxis.

### Identification of retinal in mouse spermatozoa

**Figure 2:**
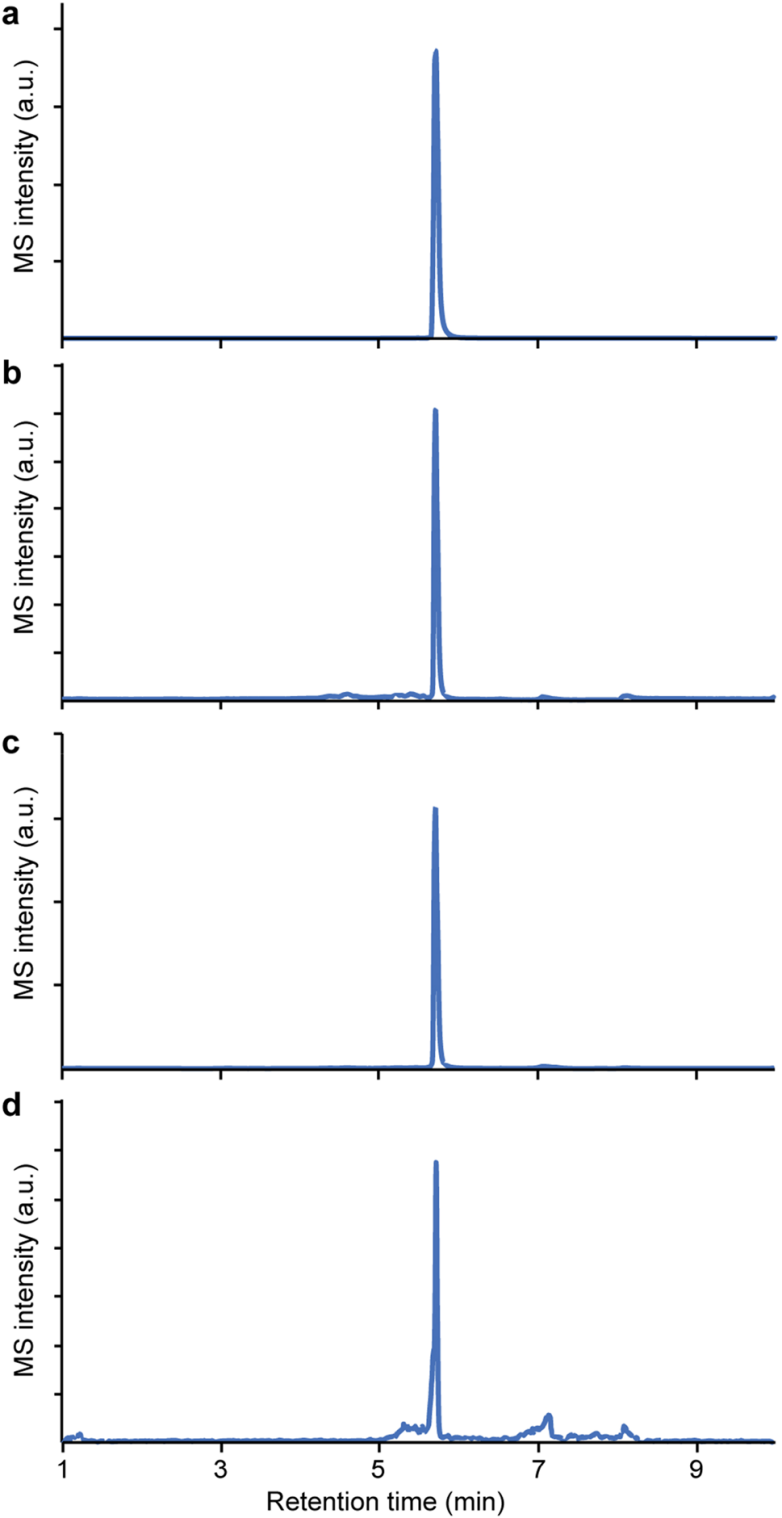
Detection of retinal by reversed-phase UPLC-MS/MS: (**a**) All-*trans* retinal (retention time 5.7 min). (**b**) An extract of mouse spermatozoa. (**c**) An extract of mouse retina. (**d**) An extract of human spermatozoa.

In view of the above results, which suggested the presence of retinal in mouse spermatozoa, we examined whether retinal can be detected in these spermatozoa. Employing reversed-phase ultra-performance liquid chromatography coupled with tandem mass spectrometry (UPLC-MS/MS), we compared extracts of mouse spermatozoa and mouse retina, the latter known to contain retinal^6^. Retinal was clearly detected in both extracts (Fig. 2).

**Figure 3:**
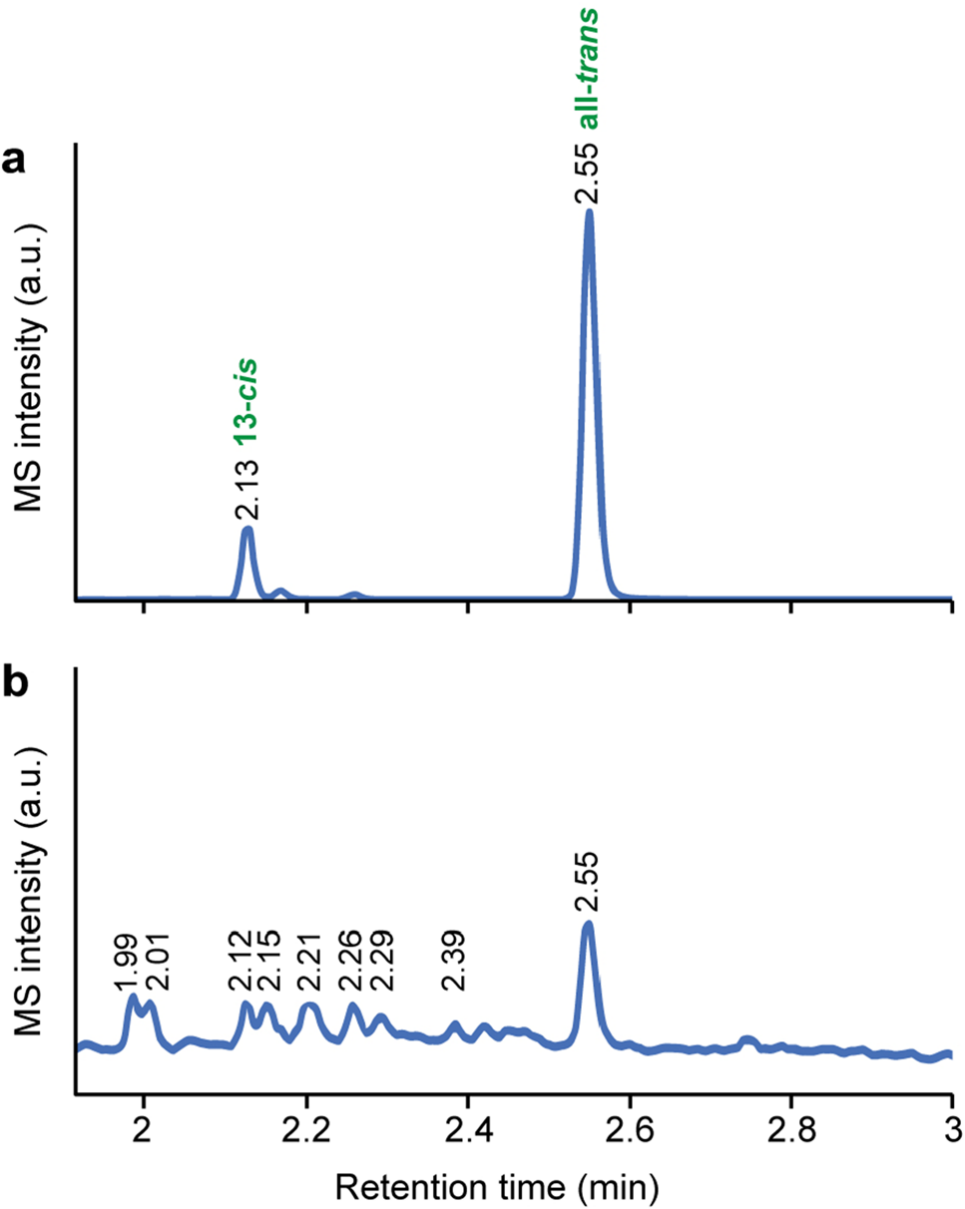
Detection of retinal isomers by UPC^2^-MS/MS in mouse spermatozoa. (**a**) All-*trans* retinal standard. (**b**) An extract of mouse spermatozoa.

**Figure 4:**
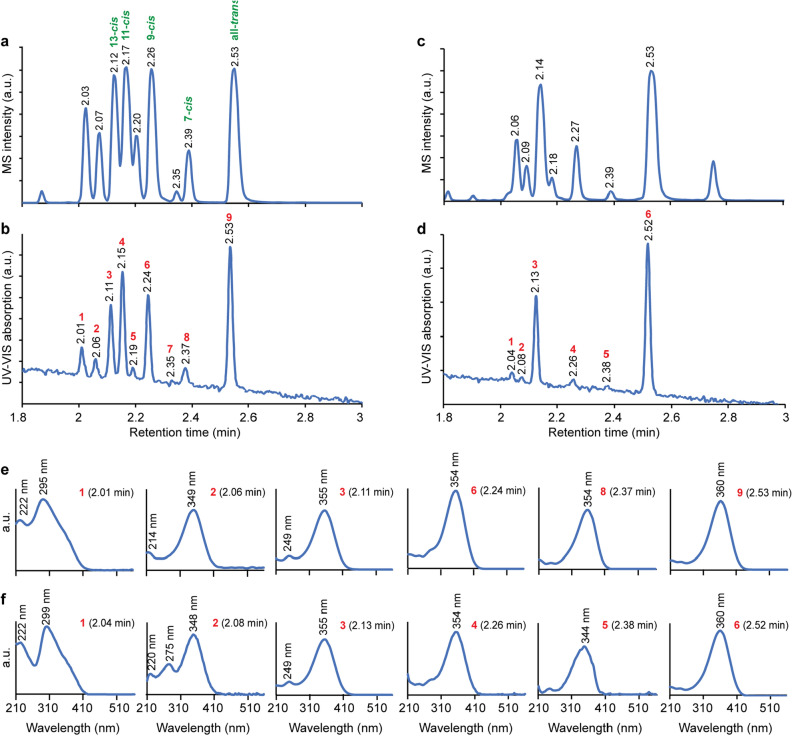
Detection of retinal isomers by UPC^2^-MS/MS in human spermatozoa. (**a**) UPC^2^-MS/MS of an irradiated all-*trans* retinal sample, known to consist of a mixture of retinal isomers. The numbers above the elution peaks indicate the retention time in minutes of each. (**b**) Relative UV–VIS absorption intensities of the peaks shown in panel a at the wavelength of maximal absorption (see panel e). The numbers above the peaks indicate the retention time. The red successive numbers are for peak identification. (**c**) As in panel a but for human sperm extract. (**d**) As in panel b but for human sperm extract. (**e**) UV–VIS spectra of the peaks eluted in the UPC^2^-MS/MS chromatograms, shown in panel a. Each spectrum is identified by the red peak number and the retention time of the corresponding MS peak. Peaks 4, 5, and 7 in the irradiated all-*trans* retinal sample, seen in panel b, are not shown in this panel because they were not detected in the sperm extract. These spectra are shown in supplementary Figure S5. (**f**) As in panel e but for human sperm extract, shown in panel c.

Establishing that retinal is present in mouse spermatozoa and involved in thermotaxis, we wished to identify its isomeric form. Retinal isomers were not resolvable by reversed-phase UPLC-MS/MS. Likewise, the well-established method for separating retinal isomers using normal-phase HPLC on a silica column^17^ was irrelevant for mouse sperm extracts. This is because the detection of isomers in this method is based on optical absorption, requiring relatively high analyte concentrations, unavailable in the case of mouse spermatozoa. Indeed, the sensitivity can be increased by MS, but the mobile phase solvents used for silica-based normal-phase chromatography are often 100% organic solvents like hexane, heptane, ether, or chloroform, which greatly reduce the MS sensitivity. The restricted quantity-availability of spermatozoa also prevented us from employing NMR, which, despite being excellent for structure determination of small molecules like retinal, it has 2–3 orders of magnitude lower sensitivity than MS^18^. We, therefore, attempted to identify the retinal isomers in mouse spermatozoa by UltraPerformance Convergence Chromatography coupled with tandem mass spectrometry (UPC^2^-MS/MS), which is an advanced technology that combines supercritical chromatography with UPLC for high-efficiency separations and fast-speed analysis of hydrophobic and thermally labile compounds^19^. An all-*trans* retinal standard yielded on UPC^2^-MS/MS two peaks (Fig. 3a), corresponding to the all-*trans* and 13-*cis* isomers of retinal (Supplementary Figure S2 for structures of the retinal isomers). (The all-*trans* isomer usually contains a certain amount of 13-*cis* retinal due to thermal isomerization^20^). The mouse sperm extract yielded several peaks (Fig. 3b). None of these additional peaks were observed in a control experiment, in which an all-*trans* retinal standard underwent the same extraction procedure as that of the spermatozoa (Supplementary Figure S3), indicating that these additional peaks were not due to some thermal isomerization of all-*trans* or 13-*cis* retinal during the extraction procedure. According to the all-*trans* retinal standard, the peaks at 2.55 and 2.12 min correspond to all-*trans* and 13-*cis* retinal. To identify the additional peaks, we analyzed in a UPC^2^-MS/MS an irradiated all-*trans* retinal sample, known to consist of a mixture of retinal isomers^21^. This mixture of isomers, which also contains tri-*cis* retinal isomers, can be obtained by irradiation of an *all-trans* retinal sample but not by its thermal treatment^21,22^. The irradiated sample yielded, among others, peaks at 2.53, 2.39, 2.26, 2.17 and 2.12 min (Fig. 4a), attributed to all-*trans*, 7-*cis*, 9-*cis*, 11-*cis* and 13-*cis* retinal, respectively^21^. Similar peaks were also detected in the mouse sperm extract (2.55, 2.39, 2.26, 2.15 and 2.12 min—Fig. 3b). Two additional peaks were detected at 2.07 and 2.03 min in the irradiated sample (Fig. 4a) and 2.01 and 1.99 min in the mouse sperm extract (Fig. 3b), attributed to tri-*cis* (and/or all-*cis*) retinal^22^. We note that variations in retention times are expected and regarded as normal in HPLC measurements in the range of ± 0.02–0.06 min^23^.

### Identification of retinal in human spermatozoa

At this stage, knowing that retinal is present in mouse sperm extract, we revisited human sperm extracts. Indeed, several peaks were observed (Fig. 4c). Comparing these peaks with those of the irradiated all-*trans* retinal sample (Fig. 4a) suggested the presence of several isomers in the human sperm extract as well. Notably, the ratios between the isomers in the human (Fig. 4c) and mouse (Fig. 3b) sperm extracts were different.

An advantage of working with human sperm extracts is that, unlike the case of mouse spermatozoa, enough extract can be obtained for determining the ultraviolet–visible (UV–VIS) spectra of the retinal isomers observed in the UPC^2^’s photodiode array (PDA) detector simultaneously with tandem MS retinal monitoring. These can provide valuable information for identifying the retinal isomers. The UV–VIS spectra of the UPC^2^ peaks detected in the irradiated all-*trans* retinal sample and in the human sperm extract are shown in Fig. 4e and 4f, respectively, and the relative heights of the optical absorption peaks are shown in Fig. 4b and 4d. Interestingly, the spectrum that corresponds to the peaks at 2.01 (Fig. 4e) and 2.04 min (Fig. 4f) consisted of an unusual strong blue-shifted band at 295 and 299 nm, respectively, accompanied by a shoulder at around 360 nm. Only three retinal isomers are known to exhibit such a strong, blue-shifted band. These are 7, 11, 13 tri-*cis* retinal, 9, 11, 13 tri-*cis* retinal, and all-*cis* retinal^24^. The absorption maxima of the blue-shifted bands of 7, 11, 13 tri-*cis* and all-*cis* retinal are at 289 and 287 nm, respectively, whereas that of 9, 11, 13 tri-*cis* retinal is at 302 nm^24^, very similar to the absorption maximum detected in the human sperm extract. Therefore, the peak detected at 2.04 min retention time in the human sperm extract and that absorbs at 299 nm (Fig. 4f) may be attributed to 9, 11, 13 tri-*cis* retinal. Likewise, the absorption maximum of the retinal isomer that corresponds to the peak eluted at 2.08 min was at 348 nm (Fig. 4f). The only retinal isomers that absorb below 350 nm are 7, 9, 11 and 7, 9, 13 tri-*cis* retinal (345 and 346 nm)^24^. Therefore, we suggest that the peak at 2.08 min in the sperm extract corresponds to one of these tri-*cis* isomers. A tri-*cis* peak of UPC^2^ was not detected in an extract of mouse retina, tested as a negative control (Figure S4).

## Discussion

Earlier findings demonstrated that opsins can act as both photosensors and thermosensors, depending on the cell type in which they reside^1–3,5,25^. This raised the question of what is different between photosensor and thermosensor opsins that enables them to sense so dissimilar stimuli. The apparently most trivial possibility that comes to mind is that they have different prosthetic groups, photosensitive and thermosensitive, respectively. If so, it would be expected that retinal, which is the photosensitive chromophore in photosensor opsin, would be substituted for some thermosensitive group in thermosensor opsin. Such a possibility predicts that opsins in spermatozoa should not contain the chromophore retinal, or if they do—retinal should not be involved in thermotaxis. The results of the current study abrogate both predictions. Thus, starvation of mice for vitamin A (i.e., for retinal) resulted in specific loss of thermotactic activity with essentially no effect on motility or on the physiological state of the cells, and replenishment of the vitamin restored the thermotactic activity (Fig. 1). This indicates that retinal, or a derivative thereof, is essential for sperm thermotaxis. Moreover, the observation that the very same anti-rhodopsin antibodies, including monoclonal antibodies, recognize both visual rhodopsin and sperm rhodopsin^5^ suggests that the proteins are apparently quite similar. The same holds for other opsins^5^. This is consistent with the conclusion that the main reason for the change in stimulus sensitivity is the retinal chromophore rather than the protein. The observation that Drosophila larvae, too, exhibits impaired temperature discrimination after vitamin A starvation^4^, suggests that the involvement of retinal in thermosensing may not be restricted to mammalian spermatozoa.

The question arises how retinal can serve as a thermosensor. The activation of rhodopsin is triggered by light absorption, which isomerizes a retinal double bond. This event induces a protein conformational alteration as well as movement of protons, anions, and cations, which leads to the active form of the protein^26^. Similar protein activation can be triggered by thermal double bond isomerization. However, due to the very high energy barrier, this is not expected to occur at a physiological temperature^27^. Our results clearly indicate that the sperm extract contains tri*-cis* retinal isomers (Fig. 4), which are usually not detected in rhodopsin extracts such as retina extraction^28^ (Figure S4). The sperm extract also contains high fraction of all*-trans* retinal as well as other retinal isomers. These other isomers could serve in the spermatozoa as precursors for the tri-*cis* retinal, formed by enzymatic processes. (Enzymatic isomeric conversion of retinal is well known in both photoreceptor cells and adjacent retinal pigment epithelial cells^29^). The opposite could also occur. Thus, isomers detected in the sperm extract could be formed during the retinal extraction process due to thermal instability of the tri-*cis* retinal. It was observed that 7,11,13 tri-*cis* and 9,11,13 tri-*cis* retinal thermally isomerize to 7,13 di-*cis* retinal and to 9,13 di-*cis* retinal at 40 °C with t_1/2_ of about 2 h in hexane solution^30,31^. Thus, the possibility that some all-*trans* retinal as well as other retinal isomers are thermally formed from tri-*cis* retinal during the extraction procedure cannot be completely excluded. Nonetheless, the tri*-cis* retinal isomers are relatively the most thermally unstable isomers of retinal^30,32^, for which reason it is tempting to suggest that they are involved in sperm thermotaxis. Twisting of retinal bonds enforced by the protein binding site can further reduce the energy barrier for thermal double bond isomerization, which will trigger protein activation.

With all these being said, an intriguing question is how spermatozoa sense and react to extremely minute temperature changes (a spermatozoon can respond to temperature changes smaller than 0.0006 °C as it swims its body-length distance) over a wide temperature range^8^. This could be the involvement of additional molecules, and/or an intense amplification mechanism of the signal produced. High amplification was proposed to be provided by the postulated high supramolecular organization of opsins in the sperm cell^5^, like the organization that exists within the retina in the membrane of rod cells^33^, where it appears to be required for their photosensitivity^34^. This amplification combined with the existence of multiple opsin types in each sperm cell, each type somewhat differently distributed in the cell and associated with only one of the two signaling pathways of thermotaxis^10^, could contribute to the extreme temperature sensitivity of mammalian spermatozoa during thermotaxis. This is reminiscent of bacterial chemotaxis, where an organized cluster of receptors provides high sensitivity and signal amplification^35^.

## Methods

### Animals and starvation for vitamin A

Studies with mice were approved by the Institutional Animal Care and Use Committee of the Weizmann Institute of Science. The methods were carried out in accordance with the approved guidelines. The study is reported in accordance with ARRIVE guidelines (https://arriveguidelines.org). C57BL/6 male mice, obtained from the animal breeding center of the Weizmann Institute of Science, were housed in positive ventilated air-filtering system in groups of three to five and maintained on a 12 h light/12 h dark cycle. Non-starved and starved mice had ad libitum access to vitamin A-containing and vitamin A-deficient diet, respectively, and water. [Vitamin A-deficient diet (AIN-93G D13110Gi) was purchased from Research Diet (New Brunswick, NJ, USA) in the form of a dry pellet and stored in sealed bags at 4 °C. The vitamin A-containing diet was Harlan-Teklad 2016 global rodent dry food pellet, containing 1500 IU vitamin A/kg (Harlan-Teklad, Indianapolis, IN, USA).] Replenished mice, obtained from initially starved mice as described in the text, were transferred to vitamin A-containing diet along with additional oral administration of vitamin A (Vitasorb A, Biocare, Redditch, UK) (725 IU/day using gavage with an olive-tip curved feeding needle) to allow sufficient daily intake of vitamin A^36^.

### Sperm retrieval and handling

Mice were sacrificed by cervical dislocation. Their caudal epididymis was removed, and spermatozoa thereof were extracted and suspended in a droplet of HTF medium containing BSA (1% w/v) under mineral oil. Subsequently, the samples were incubated for 1 h under an atmosphere of 5% CO_2_ at 37 °C for capacitation. Studies with human spermatozoa were approved by the Bioethics and Embryonic Stem Cell Research Oversight Committee of the Weizmann Institute of Science. The methods were carried out in accordance with the approved guidelines. Human semen samples were obtained from healthy donors after 3 days of sexual abstinence. Informed consent was obtained from each donor. Semen samples with normal sperm density, motility, and morphology (according to WHO guidelines^37^) were allowed to liquefy for 30–60 min at room temperature.

### Thermotaxis assays

Thermotaxis assays were carried out in a thermoseparation device^7^ as described above. The gradient tube was placed in the thermoseparation device, with the edge of the sperm-filled compartment at 35 °C and the other edge at 37 °C, creating a linear temperature gradient between these points, verified experimentally^8^. Following a 15 min separation period, spermatozoa were collected from the warmer compartment and counted using a Makler chamber. For the no-gradient control, the tube was placed in an incubator prewarmed at 35 °C. The assays were performed blindly.

### Motility analyses

Sperm cells from different diet groups were diluted to 5 × 10^6^ cells/ml in HTF containing 1% BSA. For motility recordings, sperm cells were placed in a prewarmed Makler chamber over a 37 °C Thermo Plate (Tokai Hit, Shizuoka-ken, Japan). Short videos (10–15 s each) were made using a phase-contrast Nikon Alphaphot microscope equipped with a digital camera (u-Eye, Obersulm, Germany) at 75 frames/s. The analysis was carried out by a homemade script for MatLab software. The conditions for motion analysis followed the guidelines for CASA instruments^38^.

### Determination of the fraction of capacitated spermatozoa

The fraction of capacitated spermatozoa was calculated as the difference between the fraction of acrosome-reacted cells, measured with fluorescein isothiocyanate-Pisum sativum agglutinin (FITC-PSA) or tetramethylrhodamine-Pisum sativum agglutinin (TRITC-PSA), before and after stimulation with the Ca^2+^ ionophore A23187 (dissolved in DMSO) for 30 min under an atmosphere of 5% CO_2_ at 37 °C^39–41^. As a negative control, the cells were similarly treated with DMSO instead of A23187. The stained slides were observed under a Nikon eclipse Ti-S microscope with a Nikon S Fluor 40X/0.90 NA objective (Nikon Instruments, Amsterdam, The Netherlands).

### Retinal extraction for mass spectrometry

Freshly obtained mouse semen (~ 200 µl), human semen (1.5–2 ml), and mouse retina from a single eye were used in each experiment. The samples were homogenized in 1 ml ethanol in tissue grind tube (Sz 23, Kontes, Vineland, NJ) or using pestle motor mixer (A0001, Argos Technologies, Elgin, IL) as described previously^42^. After 10-min agitation, an aqueous 15% NaCl solution was added. The suspension was vortexed and then hexane (2 ml) was added and followed by additional vortex. When phase separation was obtained, the upper (hexane) layer was separated and evaporated in nitrogen gas flow. The residue was re-dissolved in 100 µl of methanol for reversed-phase analysis or in 100 µl hexane for UPC^2^ analysis, filtered through Millex GV filter (4 mm, 0.22 µm, PVDF, Kofu, Japan), and placed in an LC–MS vial insert. The above procedure was performed at room temperature^42^ in the dark (dim red light).

### Subjecting an all-trans retinal standard to the extraction procedure

The all-*trans* retinal standard underwent the same extraction procedure. Briefly, all-*trans* retinal (10 µl from a 10 µM stock solution in chloroform) was evaporated in nitrogen flow and re-dissolved in ethanol by vortex. Hexane (2 ml) was added, followed by additional vortex. When phase separation was obtained, the upper (hexane) layer was separated and evaporated in nitrogen gas flow. The residue was re-dissolved in 100 µl hexane and filtered through Millex GV filter as described above.

### Preparation of an irradiated all-trans retinal sample

The preparation was carried out as previously reported^22^. Briefly, a solution of all-*trans* retinal (5 mg in 5 ml acetonitrile) was irradiated for 15 min with white light from a cold light source (Schott, Germany) through a > 320 nm cut-off filter. The reaction was monitored by absorption spectroscopy and HPLC. The irradiated mixture was concentrated by evaporation and was further analyzed by HPLC (Lichrosorb column; 5% ether-hexane elution solution).

### LC–MS/MS

#### Reversed-phase chromatography

Reversed-phase chromatography was performed on ACQUITY I-Class system (Waters Corporation, Milford, MA). The sample (5 µl) was injected onto an ACQUITY BEH C18 column 2.1 mm × 50 mm, 1.7 µm (Waters Corporation) kept at 30 °C. Binary gradient was applied using mobile phase A containing 5% aqueous acetonitrile with 0.1% formic acid, and mobile phase B containing 95% aqueous acetonitrile with 0.1% formic acid. The gradient elution was performed at a flow of 0.5 ml/min with initial increasing phase B from 30 to 50% B over 4.5 min, then to 100% B for 0.5 min, washing at 100%B for 2.5 min, followed by a return to initial conditions at 30% B for 0.5 min. The total run time was 10 min.

#### UPC^2^ chromatography

Supercritical chromatography was performed on an ACQUITY UPC^2^ system (Waters Corporation). The sample (2 or 10 µl) was injected onto an ACQUITY UPC^2^ HSS C18 SB column 2.1 mm × 100 mm, 1.8 µm (Waters Corporation) kept at 25 °C. Binary gradient was applied using mobile phase A containing CO_2_, and mobile phase B containing 5%-isopropanol in hexane. The gradient elution was performed at a flow of 2.5 ml/min with initial increasing phase B from 2 to 40% B over 4.5 min, then to 50% B over 0.5 min, followed by a return to initial conditions at 2% B over 0.5 min. The total run time was 7 min. Makeup solvent was 1% formic acid in 90%-aqueous methanol, flow 0.4 ml/min, CCM pressure 2000 psi.

#### Detection

A Xevo TQ-XS tandem MS (Waters Corporation) operating in positive electrospray ionization (ESI +) was used for the detection and quantification of retinal. The instrument conditions were as follows: capillary voltage 3.0 kV, source temperature 150 °C, desolvation temperature 650 °C, cone gas flow 150 l/h, and desolvation gas flow 1200 l/h. Multiple reaction monitoring (MRM) mode of acquisition was used with transitions 285.1 > 160.9 and 285.14 > 175.0 with collision energies 10 and 16 eV, respectively. Optical UV–VIS absorption spectra (210–600 nm) were recorded by a UPC^2^ PDA detector (Waters Corporation) with 2.4 nm resolution. A specific instrument configuration determined the sequential reading of absorbance followed by MS/MS chromatograms at the same acquisition run. This sometimes caused small differences in peak retention times. It should be noted, though, that the MS/MS peaks became broader than the absorption peaks due to a slight diffusion during the conversion from supercritical to liquid solution in Isocratic Solvent Manager before MS/MS detection.

### Data

All data generated or analysed during this study are included in this published article and its supplementary Data folder.

### Supplementary Information

Supplementary Information.
