# Supplementary material for: Uncommon opsin’s retinal isomer is involved in mammalian sperm thermotaxis
Source: Sci Rep. 2024 May 10;14:10699. doi: 10.1038/s41598-024-61488-3 (PMC11087470; doi:10.1038/s41598-024-61488-3)
Supplement: Supplementary file 1 — Supplementary Information. [file 41598_2024_61488_MOESM1_ESM.pdf]

SUPPLEMENTARY INFORMATION

Uncommon opsin's retinal isomer is involved in mammalian sperm thermotaxis

Alexander Brandis, Debarun Roy, Ishita Das, Mordechai Sheves and Michael Eisenbach

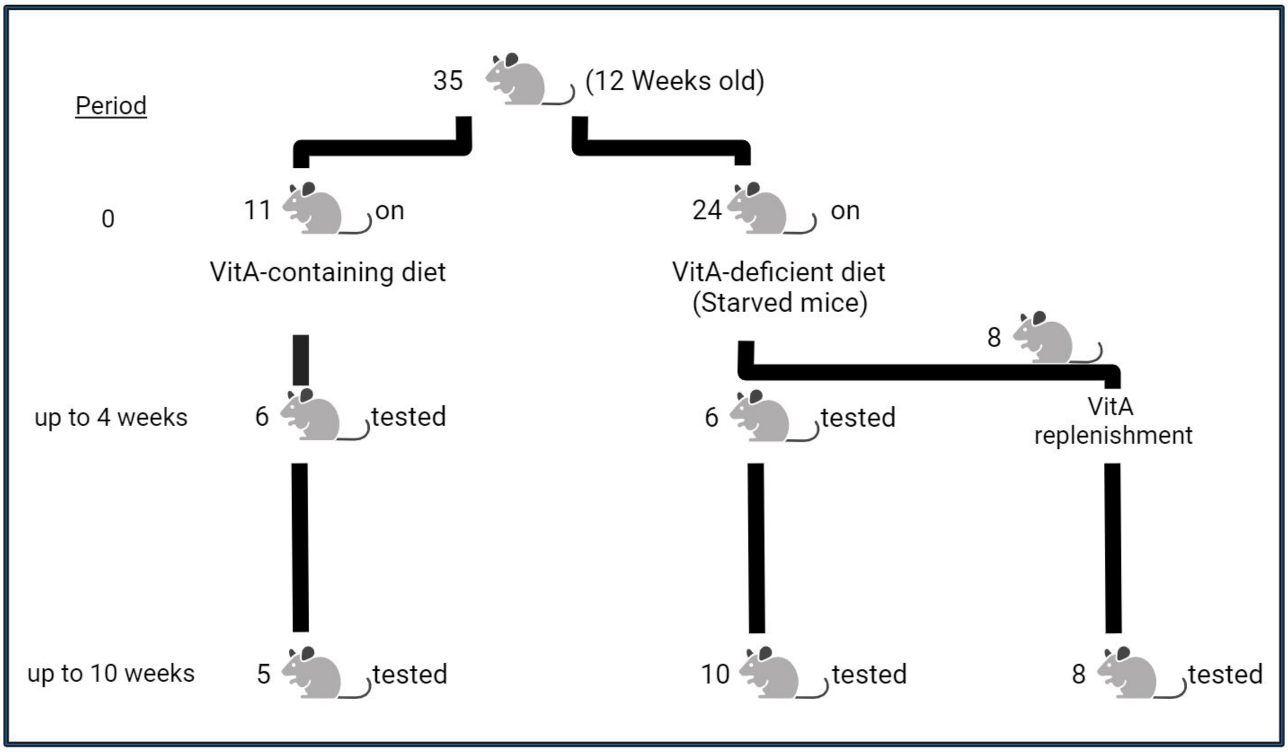

Figure S1. Vitamin A starvation procedure for Figure 1b. See text for details. The scheme was created by BioRender.

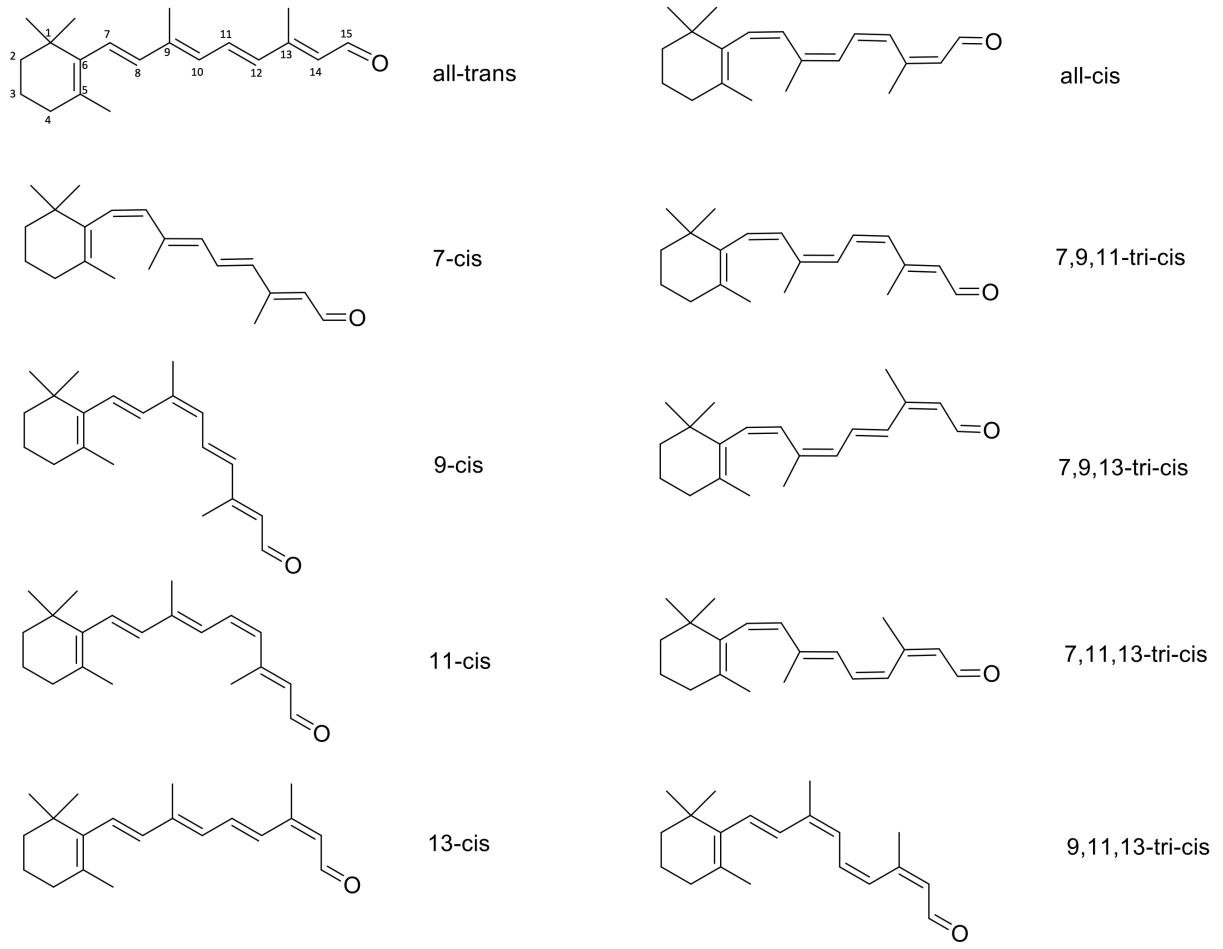

Figure S2. Structures of the retinal isomers discussed in this study.

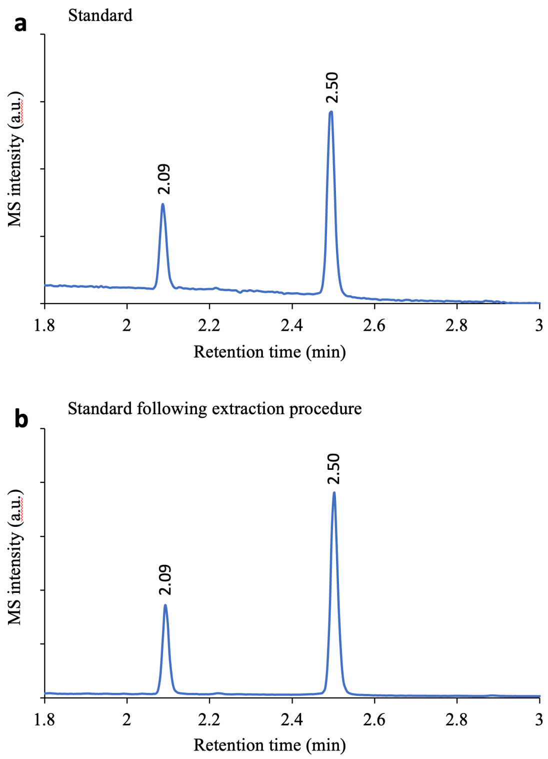

Figure S3. UPC^2-MS/MS control of all-*trans* retinal after the extraction procedure. **a,** All-*trans* retinal standard. **b,** All-*trans* retinal standard subjected to the extraction procedure.

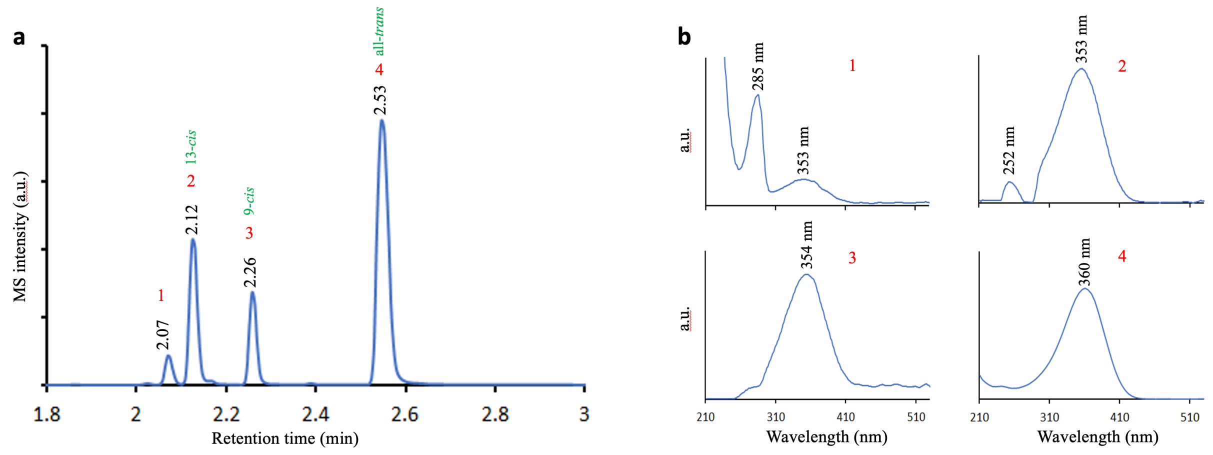

Figure S4. UPC²-MS/MS control of an extract of mouse retina. **a,** UPC²-MS/MS of mouse retina. The numbers above the elution peaks indicate the retention time in minutes of each. **b,** UV-VIS spectra of the four peaks eluted in the UPC²-MS/MS chromatograms, shown in a. Each spectrum is identified by the red peak number and the retention time of the corresponding MS peak. The peak at 2.07 min elution time (panel a), which is within the region of tri-*cis* elution time, can be identified as 7,13 di-*cis* retinal and not as tri-*cis* retinal based on its retention time and 353 nm absorption peak (panel b), in agreement with reported data[1]. Therefore, it can be concluded that the extract of mouse retina does not contain tri-*cis* retinal isomers, which were clearly detected in the human sperm extract.

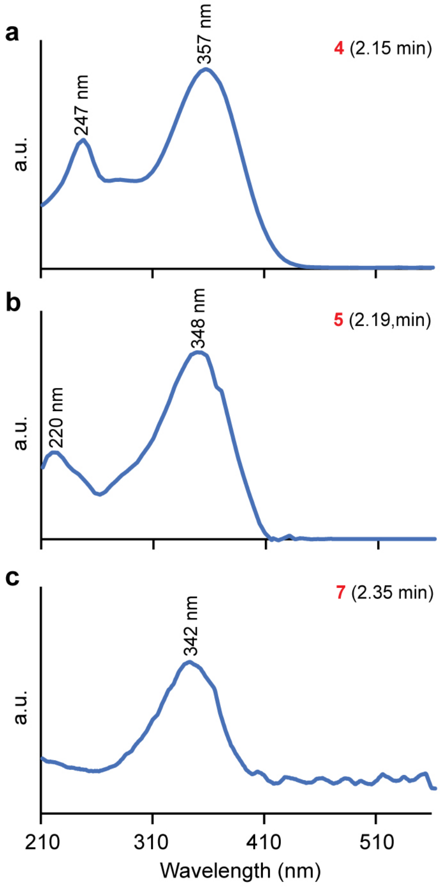

Figure S5. UV- VIS spectra of the peaks not shown in Figure 4e, eluted in the UPC²-MS/MS chromatograms of the irradiated all-*trans* retinal sample. Each spectrum is identified by the red peak number and the retention time of the corresponding MS peak.

Group	VCL	VSL	VAP	LIN	WOB	Fractal Dimensions	Mot %
Starved	261±10	70±2	109±5	0.65±0.02	0.44±0.01	1.46±0.01	54±5
Non starved	266±9	73±7	120±3	0.62±0.04	0.46±0.01	1.46±0.02	58±7
Replenished	259±8	70±3	111±4	0.65±0.03	0.45±0.02	1.44±0.01	46±3

Table S1. Motility parameters of different diet-group mice at the time of the thermotaxis assay. Swimming tracks of spermatozoa, placed on a 37°C plate, were recorded for 15 s (75 frames/s). Around 10 such video movies (each consisting of 10-50 spermatozoa) were recorded from each mouse and then analysed and averaged for each group. The values shown are mean ± SEM of N = 10, 8 and 9 starved, non-starved and replenished mice, respectively. All the parameters were found statistically indifferent between the three groups of mice ($P>0.05$ according to one way ANOVA with Tukey-Kramer post-test). Abbreviations: VCL, curvilinear velocity (time-averaged velocity of a sperm head along its actual curvilinear path, expressed in μm/s); VAP, average path velocity (velocity over an average path generated by a roaming average; μm/s); VSL, straight-line velocity (the time-average velocity of the sperm head along a straight-line from its first position to its last position; μm/s); LIN, linearity (defined as VSL/VAP); WOB, wobble (defined as VAP/VCL); FD, fractal dimension; Mot %, percentage of motile spermatozoa. FD is an expression of the degree to which the sperm trajectory fills a plane[2]. If the trajectory is a straight line with no deviations, its FD value is 1.0 because it is only in the first dimension (length). If, however, the trajectory is meandering, as in the case of a hyperactivated spermatozoon, it covers more of the plane and it has a value closer to 2.0. The FD values of most of the spermatozoa in a microscope field are between 1.0 and 2.0, although a few spermatozoa may have values larger than 2.0[3]. FD is thus used as a measure of the intensity of hyperactivation[1].

1. Liu, R. S. H. & Asato, A. E. Photochemistry and synthesis of stereoisomers of vitamin A. *Tetrahedron* **40**, 1931–1969 (1984).

2. Mortimer, S. T., Swan, M. A. & Mortimer, D. Fractal analysis of capacitating human spermatozoa. *Human reproduction (Oxford, England)* **11**, 1049–1054 (1996).

3. Katz, M. J. & George, E. B. Fractals and the analysis of growth paths. *Bulletin of Mathematical Biology* **47**, 273–286 (1985).
